# Blood groups in Native Americans: a look beyond ABO and Rh

**DOI:** 10.1590/1678-4685-GMB-2020-0255

**Published:** 2021-04-19

**Authors:** Mirelen Moura de Oliveira Rodrigues, Gabriela Höher, Gabriela Waskow, Mara Helena Hutz, Juliana Dal-Ri Lindenau, Maria Luiza Petzl-Erler, Sidia Maria Callegari-Jacques, Silvana Almeida, Marilu Fiegenbaum

**Affiliations:** 1 Universidade Federal de Ciências da Saúde de Porto Alegre (UFCSPA), Programa de Pós-Graduação em Biociências, Porto Alegre, RS, Brazil.; 2 Universidade Federal do Rio Grande do Sul (UFRGS), Departamento de Genética, Porto Alegre, RS, Brazil.; 3 Universidade Federal de Santa Catarina (UFSC), Departamento de Biologia Celular, Embriologia e Genética, Florianópolis, SC, Brazil.; 4 Universidade Federal do Paraná (UFPR), Departamento de Genética, Curitiba, PR, Brazil.; 5 Universidade Federal do Rio Grande do Sul (UFRGS), Departamento de Estatística, Porto Alegre, RS, Brazil.; 6 Universidade Federal de Ciências da Saúde de Porto Alegre (UFCSPA), Departamento de Ciências Básicas da Saúde, Porto Alegre, RS, Brazil.

**Keywords:** Genotyping, Native American population, blood group variability

## Abstract

The study presents comparisons between blood group frequencies beyond ABO and Rh blood systems in Native American populations and previously published data from Brazilian blood donors. The frequencies of Diego (c.2561C>T, rs2285644), Kell (c.578C>T, rs8176058), Duffy (c.125A>G, rs12075, c.1−67T>C, rs2814778) and Kidd (c.838A>G, rs1058396) variants in Kaingang (n=72) and Guarani (n=234) populations from Brazil (1990-2000) were obtained and compared with data from these populations sampled during the 1960s and with individuals of different Brazilian regions. Data showed high frequencies of DI*01 and FY*01 alleles: 11.8% and 57.6% in Kaingang and 6.8% and 75.7% in Guarani groups, respectively. The main results indicated: (1) reduction in genetic distance over time of Kaingang and Guarani in relation to other Brazilian populations is suggestive of ongoing admixture; (2) significant differences in some frequencies of blood group markers (especially Diego, Kidd and Duffy) in relation to Native Americans and individuals from different geographical regions of Brazil. Our study shows that the frequency of red blood cell polymorphisms in two Native American groups is very different from that of blood donors, when we evaluated blood groups different from ABO and Rh systems, suggesting that a better ethnic characterization of blood unit receptors is necessary.

## Introduction

Brazil has more than 207 million inhabitants and is one of the most diverse countries in the world as a result of successive migratory waves (European, African and Asian) in addition to the Native American populations already residing in Brazilian territory prior to colonization (Leite et al., 2009; IBGE, 2017). The Guarani are the most populous Brazilian Native American population, and they reside in the states of Rio Grande do Sul, Santa Catarina, Paraná, São Paulo, Rio de Janeiro, Espírito Santo and Mato Grosso do Sul, as well as in other Latin American countries (Argentina, Paraguay, Bolivia and Uruguay) (Comissão de Cidadania e Direitos Humanos, 2010). The Kaingang are the third most populous Native American group in Brazil; the Kaingang live in the states of São Paulo, Paraná, Santa Catarina and Rio Grande do Sul (Lindenau et al., 2016). Although Brazil harbors a significant proportion of the Native Americans in South America, only 0.4% (896,000 people) of the total Brazilian population self-classify themselves as indigenous (‘‘indígena’’) (IBGE, 2012a). However, this percentage represents an undercount, given that this figure does not include several Brazilian Native American communities that live in “isolated populations” and that some ethnic groups are in the process of ethnic reaffirmation following years of domination and cultural repression (Luciano, 2006; IBGE, 2012b). As a number of these populations have remained in their precolonial state, they are geographically and culturally isolated and have close links with their territories; thus, they have well-defined social, economic and political systems, beliefs, languages and cultures (Luciano, 2006). Reproductive isolation may produce populations with genetic pools that differ from those of populations living in panmixia.

The International Society of Blood Transfusion (ISBT) currently recognizes 366 blood group antigens, of which 328 are dispersed within 38 blood group systems (ISBT, 2020); some of these antigens cause alloimmunization. Alloimmunization can lead to hemolytic transfusion reactions (HTRs) and hemolytic diseases of the fetus and newborn (HDFNs) (Alves et al., 2012). These adverse effects occur more frequently when blood donor recipients or parents of a child differ in their genetic makeup as a result of diverse ethnicities (Kenny, 2006; Saleh et al., 2018). ABO and Rh are the most important blood group systems in transfusional medicine; however, other clinically significant systems are worthy of consideration, such as the Kell, Duffy, Diego and Kidd blood systems (Westhoff, 2019).

The blood group allele and phenotype frequencies change according to ethnic group (Romphruk *et al*., 2019) due to selection (as with Duffy alleles), genetic drift and other evolutionary factors. In some populations, blood group alleles could be considered to be an anthropological marker, such as the Di^a^ antigen for Native Americans (Bégat et al., 2015). In blood centers, ABO and RhD phenotypes are identified for all blood donations to avoid HTR (Ministério da Saúde, 2017). However, it is also important to search for other blood systems, such as Kell, Duffy, Kidd, Diego and other blood group antigens, because they could also be implicated in HTR and DHFN (Poole and Daniels, 2007). The investigation of genetic polymorphisms of other blood groups in Native Americans is important for determining whether the allele frequencies of blood groups in a focal population differ from those of blood donors from the general population. In addition, these studies may provide useful information to blood centers regarding focal ethnic groups.

The aims of this study were: (a) to determine the frequency of several clinically important blood group alleles, that is, Diego (*c.2561C>T* - DI*01/*02 - rs2285644), Kell (*c.578C>T* - KEL*01/*02 - rs8176058), Duffy (*c.125A>G* - FY*01/*02 - rs12075 and *c.1−67T>C* - FY*02N.01/*02 - rs2814778) and Kidd (*c.838A>G* - JK*01/*02 - rs1058396), in two Native American populations from Brazil (the Kaingang and Guarani) and; (b) to compare the observed frequencies with published data from individuals residing in different Brazilian regions, as well as with published data that were obtained from the Kaingang and Guarani populations in the 1960s.

## Subjects and Methods

### Sample characterization

We analyzed data from 306 Native American individuals: 72 Kaingang and 234 Guarani (154 from the Kaiowá subdivision and 80 from the Ñandeva subdivision), which were sampled between 1990 and 2000. In Table 1, we present the characteristics of the populations (the name of the locality, geographical coordinates and sampling period). This study was approved by the Research Ethics Committee of the Universidade Federal de Ciências da Saúde de Porto Alegre (UFCSPA) (N°1.885.647).

**Table 1 ‒:** Geographic location and year of collection of the samples studied.

	Population	
Characteristics	Guarani	Kaingang
Localities	Amambai/MS Limão Verde/ MS Porto Lindo/ MS	Nonoai/ RS
Geographical coordinates	55°12’W, 23°6’S 55°6’W, 23°12’S 54°30’W, 23°48’S	52°45’W, 27°20’S
Sampling period	1992 - 1993	2000

Table adapted from Lindenau et al. (2016).

### DNA extraction and genotyping

Genomic DNA was extracted from peripheral blood leukocytes by a standard salting-out procedure (Lahiri and Numberger, 1991). DNA samples were quantified by optical density at 260 nm (BioSpec-Nano, Shimadzu, Columbia, MD) and diluted to 10 ng/µL.

The genotypes of the 5 polymorphisms of blood groups were determined by allele discrimination using a hydrolysis probe with TaqMan 5′-nuclease assays on a real-time PCR system (StepOnePlus, Applied Biosystems, Foster City, CA, USA). The following assays were used (Thermo Fischer Scientific, Waltham, MA): C__26654865_10 (DI*01/*02 - rs2285644), AHABI4V (KEL*01/*02 - rs8176058), C___2493442_10 (FY*01/*02 - rs12075), C__15769614_10 (FY*02N.01/*02 - rs2814778) and C___1727582_10 (JK*01/*02 - rs1058396). The reactions were performed with fast thermal cycling conditions, and the reagent concentrations were as follows: 10 ng of DNA, 1X TaqMan genotyping assay, 1X TaqMan genotyping master mix and nuclease-free water.

### Data collection of previously published blood group variants

Genotypic data of the samples of the present study were compared to phenotypic data obtained from 214 Kaingang (Ligero, Guarita, Nonoai and Cacique Doble, Rio Grande do Sul state) and 34 Guarani (Chapecó and Duque de Caxias, Santa Catarina state) samples collected in the 1960s (Salzano, 1964a, 1964b; Salzano *et al*., 1980) (Table S1).

In addition, we reviewed previous studies of blood groups from other regions of Brazil, determined the allele frequency of the investigated polymorphisms and compared these data with those of the Kaingang and Guarani groups. Data on allele frequencies from blood donors were collected from (1) the South region of Brazil (by state): Rio Grande do Sul (RS, n = 407) (Waskow *et al*. 2020), Santa Catarina (SC, n = 373) (Costa et al., 2016) and Paraná (PR, n = 251; Brazilian Japanese descendants, n = 209) (Flôres et al., 2014; Zacarias et al., 2016); (2) the Southeast region: São Paulo (SP, n = 948) (Ribeiro et al., 2009); and (3) the Northeast region: Bahia (BA, n = 196) (Costa *et al.,* 2016). The following methodologies were employed for blood group genotyping in these studies: restriction fragment length polymorphism - polymerase chain reaction (RFLP-PCR) (Flôres *et al.,* 2014; Costa *et al*., 2016; Zacarias *et al.,* 2016), DNA array analysis performed with the human erythrocyte antigen (HEA) BeadChip (Ribeiro *et al.,* 2009) and real-time PCR (Waskow *et al.,* 2020).

### Statistical analyses

Allele frequencies were estimated from our data by gene counting. We estimated allele frequencies of data collected from the literature using $q=\sqrt{homozygote}$. For our data, Hardy-Weinberg equilibrium (HWE) was tested for each locus using an analog of Fisher´s exact test, described in Guo and Thompson (1992). Genetic diversity between populations was evaluated using a chi-square test, while Bonferroni correction was employed to adjust for multiple comparisons. These analyses were performed in Arlequin v.3.5 (Excoffier and Lischer, 2010). Pairwise Nei’s genetic distances (Nei et al., 1983) were employed to estimate DA genetic distances between populations using Poptree2 (Takezaki et al., 2010). A nonmetric multidimensional scaling (MDS) for DA distances was performed to visualize the populations in a two-dimensional frame. A stress (distortion) lower than 0.05 was considered to be acceptable. To conduct MDS, we employed the Statistical Package for Social Sciences Version 18.0 software (SPSS, Chicago, IL). For the statistical tests, a p-value < 0.05 was considered to be significant.

## Results

The Guarani cultural-linguistic subdivisions Ñandeva and Kaiowá did not exhibit significant differences in allele frequencies; thus, we clustered these two subdivisions in a group called Guarani (data not shown). The observed genotype distributions in the Kaingang and Guarani ethnic groups were in HWE for all systems. The comparison between Kaingang and Guarani differed in the frequencies of the FY*01, FY*02 and FY*02N.01 alleles (*p* = 0.00175 ± 0.0021).

Gene frequencies were compared between Kaingang, Guarani and other Brazilian samples (Table 2). We observed significant differences in allele frequencies when Kaingang and Guarani people were compared with non-native Brazilian individuals from the South, Southeast and Northeast regions. For the Kaingang group, all polymorphisms exhibited significant differences, except for Kell. The primary differences were in the Duffy and Kidd systems. The Guarani also differed from other populations in the Duffy and Kidd systems. The Diego system also exhibited differences between Native Americans and other populations. Despite these differences, Guarani showed a similar genetic profile with Brazilian-Japanese descendants, with differences being observed only in the Kidd system.

**Table 2 -:** Minor allele frequencies of blood groups studied in Kaingang and Guarani compared with other Brazilian data.

	Present study		South				Southeast	Northeast
	Kaingang (n= 72)	Guarani (n= 234)	RS (n= 407)	SC (n= 373)	PR (n= 251)	PR-BJD (n= 209)	SP (n= 948)	BA (n= 196)
Diego								
DI*01	0.118	0.069	0.010*†	0.028*†	0.022*†	0.043*	0.020*†	0.015*†
Kell								
KEL*01	0	0.002	0.005	0.031†	0.044†	0.002	0.024†	0.020
Duffy								
FY*01	0.576†	0.757*	0.404†	0.405*†	0.436†	0.784*	0.360*†	0.247*†
FY*02	0.376†	0.231*	0.490†	0.541*†	0.540†	0.205*	0.455*†	0.317*†
FY*02N.01	0.048†	0.012*	0.106†	0.054*†	0.024†	0.011*	0.185*†	0.436*†
Kidd								
JK*01	0.326	0.407	0.560*	0.531*	0.502*	0.538*†	0.460*	0.380
JK*02	0.674	0.593	0.440*	0.469*	0.498*	0.462*†	0.540*	0.620

RS: Blood donors from the state of Rio Grande do Sul (Waskow *et al*., 2020)

SC: Blood donors from the state of Santa Catarina (Costa *et al*., 2016)

PR: Blood donors from the Southwest region of the state of Parana (Zacarias *et al*., 2016)

PR-BJD (Brazilian Japanese descendants): Blood donors from the state of Parana (Flôres *et al*., 2014)

SP: Blood donors from the state of São Paulo (Ribeiro *et al*., 2009)

BA: Mixed population from the state of Bahia (Costa *et al*., 2016)

* Kaingang with other studies, p < 0.05

† Guarani with other studies, p < 0.05

**Figure 1 -:**
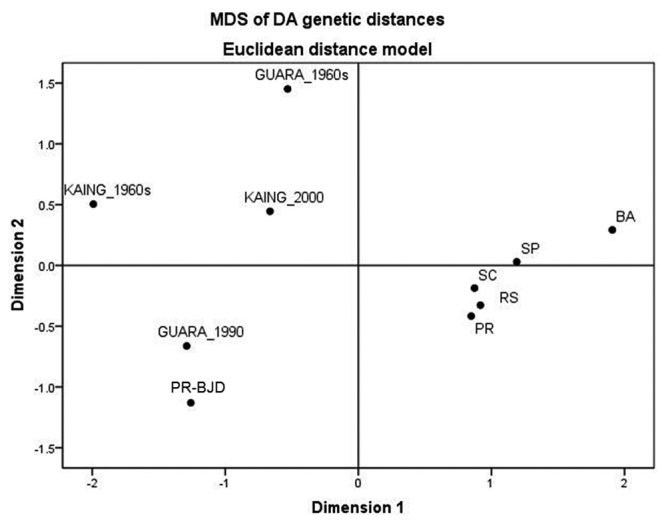
Nonmetric multidimensional scaling of DA genetic distances between 10 population samples, based on Diego, Kell and Duffy red cell polymorphisms (stress = 0.04; R2 = 0.99). Legend: GUARA_1990: Guarani present study, KAING_2000: Kaingang present study, GUARA_1960s: Guarani sampled in the 1960s (Salzano 1964a), KAING_1960s: Kaingang sampled in the 1960s (Salzano *et al*., 1980), RS: Blood donors from the state of Rio Grande do Su l (Waskow *et al*., 2020), SC: Blood donors from the state of Santa Catarina (Costa *et al*., 2016), PR: Blood donors from the Southwest region of the state of Parana (Zacarias *et al*., 2016), PR-BJD (Brazilian Japanese descendants): Blood donors from the state of Parana (Flôres *et al*., 2014), SP: Blood donors from the state of São Paulo (Ribeiro *et al*., 2009), BA: Mixed population from the state of Bahia (Costa *et al*., 2016).

We also computed pairwise DA genetic distances between Kaingang and Guarani samples collected in this study, samples obtained from native groups from the 1960s, and samples from the previously mentioned present-day Brazilian populations in the analysis. Multidimensional scaling was applied to DA distances to produce Figure 1. The median DA genetic distance (0.017) between the Kaingang sampled for this study (Kaingang 2000) and samples of six other Brazilian populations was smaller than that observed for samples from the 1960s (Kaingang 1960, median DA = 0.044). The same tendency was observed for the Guarani, but the decrease was considerably less pronounced (median DA for Guarani 1990 = 0.026; Guarani 1960 = 0.028). This pattern did not change when Japanese descendants were omitted, or when the analysis included exclusively non-native individuals of the South region. From the analysis of allele frequencies, we observed a temporal decrease in the DI*01 allele, which is a well-known Native American marker, in both Native American populations (Kaingang 1960s: 0.240; Kaingang 2000: 0.118; Guarani 1961-1963: 0.233; Guarani 1992-1993: 0.069). In addition, the genetic marker of European ancestry KEL*01 was only observed in Guarani, which were recently sampled (1992-1993), at a prevalence of 0.002. We also observed a dual temporal effect on the FY*01 allele frequency: for the Kaingang sampled in Rio Grande do Sul in the 1960s (Salzano *et al*., 1980), and there was a reduction in allele frequency (0.710 vs. 0.576), while an increase was observed between Guarani from Rio Grande do Sul relative to Santa Catarina in the 1960s (Salzano, 1964a) (0.450 vs. 0.757).

## Discussion

The frequencies with which blood group antigens are detected differ based on the studied population. For example, the Di^a^ antigen (DI*01 allele), which is considered an anthropological marker of Native American populations, was observed at differing frequencies in the studied population (Daniels, 2002). Knowledge of the different frequencies of RBC (red blood cell) polymorphisms among populations is useful for understanding anthropology and is helpful for transfusion medicine (Costa et al., 2016). Several studies have been conducted with Native American ethnic groups to characterize their genetic variability (Salzano, 1964a; Salzano *et al.* 1997, 1980; Hünemeier et al., 2012; Reich et al., 2012; de Souza and Santos, 2014; Bégat et al., 2015; Lindenau et al., 2016); however, no study has evaluated variations in blood groups along with relevant contexts from a clinical perspective.

Our primary results indicated the following: (1) Kaingang and Guarani groups differ only in the Duffy system; (2) the reduction of genetic distance over time, which was exhibited Kaingang and Guarani relative to other Brazilian populations, is suggestive of historical admixture; (3) nevertheless, there are significant differences regarding some frequencies of blood group markers (especially Diego, Kidd and Duffy) between Native Americans and individuals from different geographical regions of Brazil.

The Brazilian population is highly heterogeneous due to genetic admixture between several ancestral groups, primarily Native Americans, Europeans and Africans (Suarez-Kurtz et al., 2012). The relative contributions of the three main parental populations vary throughout the country. Data from a study of 934 Brazilian individuals, self-categorized as having white, brown and black color, showed that European genomic ancestry is 0.601 in the Northeast region, 0.742 in the Southeast region and 0.795 in the South region; African ancestry is 0.293, 0.173 and 0.103 in the Northeast, Southeast, and South regions, respectively; and Native American ancestry is 0.089, 0.073 and 0.094 in the Northeast, Southeast, and South regions, respectively (Pena et al., 2011). These observations have important implications for transfusion medicine, especially when blood donors are not matched to the recipient of the blood unit or when patients are polytransfused. Native Americans are a unique population with high genetic diversity and a history of genetic isolation (Lindenau et al., 2016). If the recipient were Native American, the blood bank may have difficulties locating a compatible blood unit. The Native American contribution is higher in the North (0.185) than in other regions, but it is not negligible in the South (0.094) *(* Pena *et al.,* 2011). We observed significant differences in the frequencies of all genetic markers in our study compared with non-native Brazilian subjects, and these markers can cause immune reactions (Table 2).

**Figure 2 -:**
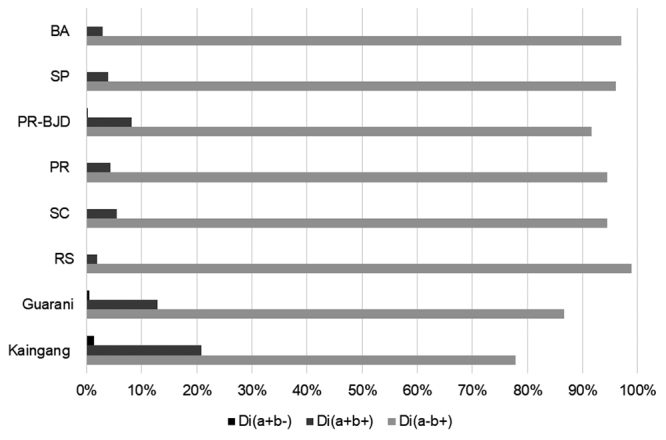
Phenotype frequencies by Diego blood group alleles. Legend: Guarani: present study Kaingang: present study, RS: Blood donors from the state of Rio Grande do Sul (Waskow *et al*., 2020), SC: Blood donors from the state of Santa Catarina (Costa *et al*., 2016), PR: Blood donors from the Southwest region of the state of Parana (Zacarias *et al*., 2016), PR-BJD (Brazilian Japanese descendants): Blood donors from the state of Parana (Flôres *et al*., 2014), SP: Blood donors from the state of São Paulo (Ribeiro *et al*., 2009), BA: Mixed population from the state of Bahia (Costa *et al*., 2016).

DI*01 has often been associated with HDFN, but it can also cause HTR (Byrne and Byrne, 2004). There are three possible genotypes to predict three possible phenotypes: Di(a+b-) (DI*01/DI*01 genotype), Di(a+b+) (DI*01/DI*02 genotype) and Di(a-b+) (DI*02/DI*02 genotype) (Flôres et al., 2014). As the DI*01 allele is an anthropological marker of Native American populations (Bégat et al., 2015) and it is infrequently observed in other populations, there is a low probability of locating a compatible donor for the Di(a+b-) phenotype. Brazil has a national program of rare blood donors where Di(a+b-) blood donors are available. The DI*01 allele frequency was observed to 11.8% in Kaingang and 6.8% in Guarani. These frequencies are considerably higher than all other published data for the Brazilian population (frequency range: 0.9%-4.3%) (Cavasini et al., 2007; Ribeiro et al., 2009; Flôres *et al.,* 2014; Latini et al., 2014; Costa et al., 2016; Zacarias et al., 2016; Martins et al., 2017). Exceptions are Brazilian-Japanese populations, which had a frequency (4.3%) similar to that of the Guarani (6.9%) (Table 2). Therefore, there is a low probability of finding a compatible donor for the Di(a+b-) phenotype among non-native Brazilian donors (Figure 2), although the probability is slightly higher among people with Native American (when possible) and Japanese ancestry. From the allele frequencies obtained in the present study, it is possible to calculate the probability of finding an individual with a Di(a+b-) phenotype: 1.39% (14/1,000 individuals) among the Kaingang and 0.47% (5/1,000 individuals) among the Guarani. Considering data from published articles, the lowest probability of finding an individual with a Di(a+b-) phenotype is among blood donors from Rio Grande do Sul (0.0081%, that is, 8/100,000 individuals). The highest probability of finding a compatible donor of this profile is in Japanese descendants of Paraná (0.18% or 2/1,000 individuals). Among samples from other blood donors, the highest probability was observed in Santa Catarina state with 9/10,000 individuals (0.09%).

According to Bégat et al. (2015), the frequency of the DI*01 allele is considerably higher in individuals who speak Gê (17.1%) and Tupi (18.4%) (Bégat *et al*., 2015) than in Brazilian blood donors. These data are consistent with our results: the DI*01 allele occurs at a frequency of 11.8% in the Kaingang (who speak the Gê language) and 6.8% in the Guarani (from the Tupi language family). The allele frequency found in our study is slightly lower than those proposed by Bégat *et al.* (2015) for the DI*01 allele in Native Americans. In addition, the temporal comparison between allelic frequencies of blood groups evaluated in the Kaingang and Guarani indicates a decrease in the DI*01 allele frequency, which may be explained by different reasons. First, although the samples collected in the 1960s and more recently were obtained from the same Native American groups, the sampled population is not exactly the same; therefore, the subjects could have had different allele frequencies. Moreover, these results may also suggest that there is a continuing process of admixture among these populations with non-Native Americans; perhaps modifications in the culture of marriages with non-Native Americans may have happened more recently in these ethnic groups. The increased frequency of the FY*02 (Fy^b^) allele in Kaingangs and the detection of the KEL*01 (K) allele in the Guarani population suggest that admixture may partially explain these results (Table S1).

The KEL*01 allele (K antigen) is the third most potent allele for triggering alloimmunization implicated in potential HTR and HDFN (Flôres et al., 2014), and in relation to immunogenicity, only the D antigen is considered more immunogenic (Denomme, 2015). In HDFN, anti-K induces mild hyperbilirubinemia and reticulocytopenia. In addition, the blocking phenomenon, which is a false negative typing of the fetal cells’ K antigen, can be caused by a high anti-K titre in the maternal blood (Manfroi and Velati, 2017). Therefore, this allele is important for determining the presence of the K antigen when there is a blood transfusion in Kaingang and Guarani individuals, given that the K antigen is present at a considerably lower frequency in these populations than among non-native blood donors. K-negative antigen (KEL*02/*02 genotype) has been documented in other studies of native South American groups (Salzano, 1964a).

Duffy blood group antigens (Fy^a^ - FY*01 allele and Fy^b^ - FY*02 allele) are associated with immediate and delayed HTR. The Duffy glycoprotein is a receptor in the erythrocyte membrane to chemokines and plays a role as a portal to malaria pathogens in RBCs. Allele FY*02N.01, which is caused by the point mutation *c.1-67T>C* (rs2814778) in the 5’ untranslated region, prevents Fyb antigen expression exclusively in red blood cells and might prevent malaria infection in some people (Höher et al., 2017). The frequencies of the FY*02N.01 allele were similar in the Kaingang and some southern Brazilian populations (RS and PR) and in Guarani and Brazilian-Japanese descendants. However, in other Brazilian populations, the frequencies were higher due to an increased African contribution to the Brazilian population.

To the best of our knowledge, the literature does not provide data showing how many Native Americans undergo blood transfusions in Brazil. However, this screening is important in the process of locating the most appropriate blood unit in case one is needed. This study demonstrates that variability in RBC polymorphisms (DI*01/*02, KEL*01/*02, FY*01/*02, FY*02N.01/*02, JK*01/*02) in Kaingang and Guarani populations is different from that of the RBCs of blood donors, with the exception of people of Japanese ancestry compared with people of Guarani ancestry. When blood transfusion is required for a Brazilian Native American and a Native American or their descendants are not available to donate, based on our data and those presented in the literature, we suggest that blood centers recruit blood donors with Asian ancestry. In addition, we recommend that additional studies of the descendants of Native American populations be conducted to help to elucidate the diversity of the Brazilian population.

## Supplementary material

The following online material is available for this article:

Table S1 - Allele and estimated phenotype frequencies of antigens blood group in Kaingang and Guarani groups according to year of data collection.

## References

[B1] Alves VM, Martins PRJ, Soares S, Araújo G, Schmidt LC, Costa SS de M, Langhi DM, Moraes-Souza H (2012). Pesquisa de aloimunização após transfusão de concentrados de
hemácias em um estudo prospectivo. Rev Bras Hematol Hemoter.

[B2] Bégat C, Bailly P, Chiaroni J, Mazières S (2015). Revisiting the Diego blood group system in Amerindians: Evidence
for gene-culture comigration. PLoS One.

[B3] Byrne KM, Byrne PC (2004). Other blood group systems - Diego,Yt, Xg, Scianna, Dombrock,
Colton, Landsteiner - Wiener, and Indian. Immunohematology.

[B4] Cavasini CE, de Mattos LC, Couto Á, Couto V, Gollino Y, Moretti LJ, Bonini-Domingos CR, Rossit AR, Castilho L, Machado RL (2007). Duffy blood group gene polymorphisms among malaria vivax patients
in four areas of the Brazilian Amazon region. Malar J.

[B5] Comissão de Cidadania e Direitos Humanos da Assembleia Legislativa
do Estado do Rio Grande do Sul (2010). Coletivos Guarani no Rio Grande do Sul. Territorialidade,
interetnicidade, Sobreposições e Direitos Específicos.

[B6] Costa DC, Schinaider AA, Santos TM, Schörner EJ, Simon D, Maluf SW, Moraes ACR de, Silva MCS (2016). Frequencies of polymorphisms of Rh, Kell, Kidd, Duffy and Diego
systems of Santa Catarina, southern Brazil. Rev Bras Hematol Hemoter.

[B7] Daniels G (2002). Human blood groups, second.

[B8] de Souza VS, Santos RV (2014). The emergence of human population genetics and narratives about
the formation of the Brazilian nation (1950-1960). Stud Hist Philos Sci Part C Stud Hist Philos Biol Biomed Sci.

[B9] Denomme GA (2015). Kell and Kx blood group systems. Immunohematology.

[B10] Excoffier L, Lischer HEL (2010). Arlequin suite ver 3.5: a new series of programs to perform
population genetics analyses under Linux and Windows. Mol Ecol Resour.

[B11] Flôres MALR, Visentainer JEL, Guelsin GAS, Fracasso A de S, Melo FC de, Hashimoto MN, Sell AM (2014). Rh, Kell, Duffy, Kidd and Diego blood group system polymorphism
in Brazilian Japanese descendants. Transfus Apher Sci.

[B12] Guo SW, Thompson EA (1992). Performing the exact test of Hardy-Weinberg proportion for
multiple alleles. Biometrics.

[B13] Höher G, Fiegenbaum M, Almeida S (2017). Molecular basis of the Duffy blood group system. Blood Transfus.

[B14] Hünemeier T, Gomez-Valdes J, Ballesteros-Romero M, de Azevedo S, Martinez-Abadias N, Esparza M, Sjovold T, Bonatto SL, Salzano FM, Bortolini MC (2012). Cultural diversification promotes rapid phenotypic evolution in
Xavante Indians. Proc Natl Acad Sci U S A.

[B15] IBGE (2012). O Brasil indígena.

[B16] IBGE (2012). Os indígenas no Censo Demográfico 2010: primeiras considerações com base
no quesito cor ou raça.

[B17] IBGE (2017). Resolução n. 4, de 28 de agosto de 2017. Diário Oficial da União.

[B18] ISBT (2020). Red Cell Immunogenetics and Blood Group Terminology.

[B19] Kenny MG (2006). A question of blood, race, and politics. J Hist Med Allied Sci.

[B20] Lahiri DK, Numberger JI (1991). A rapid non-enzymatic method for the preparation of HMW DNA from
blood for RFLP studies. Nucleic Acids Res.

[B21] Latini FRM, Gazito D, Arnoni CP, Muniz JG, De Medeiros Person R, Carvalho FO, Baleotti W, Castilho L, Barreto JA (2014). A new strategy to identify rare blood donors: Single polymerase
chain reaction multiplex SNaPshot reaction for detection of 16 blood group
alleles. Blood Transfus.

[B22] Leite FPN, Santos SEB, Rodríguez EMR, Callegari-Jacques SM, Demarchi DA, Tsuneto LT, Petzl-Erler ML, Salzano FM, Hutz MH (2009). Linkage disequilibrium patterns and genetic structure of
Amerindian and non-Amerindian Brazilian populations revealed by long-range
X-STR markers. Am J Phys Anthropol.

[B23] Lindenau JDR, Salzano FM, Hurtado AM, Hill KR, Petzl-Erler ML, Tsuneto LT, Hutz MH (2016). Variability of innate immune system genes in Native American
populations - Relationship with history and epidemiology. Am J Phys Anthropol.

[B24] Luciano GS (2006). O Índio Brasileiro: o que você precisa saber sobre os povos indígenas no
Brasil de hoje.

[B25] Manfroi S, Velati C (2017). K-antigen blocking in a case of haemolytic disease of the foetus
and newborn. Blood Transfus.

[B26] Martins ML, da Silva AR, Santos HC, Alves MT, Schmidt LC, Vertchenko SB, Dusse LMSA, Silva Malta MCF (2017). Duffy blood group system: New genotyping method and distribution
in a Brazilian extra-Amazonian population. Mol Cell Probes.

[B27] Ministério da Saúde (2017). Do regulamento técnico de procedimentos hemoterápicos. Portaria
Consolidação n. 5.

[B28] Nei M, Tajima F, Tateno Y (1983). Accuracy of estimated phylogenetic trees from molecular
data. J Mol Evol.

[B29] Pena SDJ, di Pietro G, Fuchshuber-Moraes M, Genro JP, Hutz MH, Kehdy F de SG, Kohlrausch F, Magno LAV, Montenegro RC, Moraes MO (2011). The genomic ancestry of individuals from different geographical
regions of Brazil is more uniform than expected. PLoS One.

[B30] Poole J, Daniels G (2007). Blood group antibodies and their significance in transfusion
medicine. Transfus Med Rev.

[B31] Reich D, Patterson N, Campbell D, Tandon A, Mazieres S, Ray N, Parra MV, Rojas W, Duque C, Mesa N (2012). Reconstructing Native American population history. Nature.

[B32] Ribeiro KR, Guarnieri MH, Da Costa DC, Costa FF, Pellegrino J, Castilho L (2009). DNA array analysis for red blood cell antigens facilitates the
transfusion support with antigen-matched blood in patients with sickle cell
disease. Vox Sang.

[B33] Romphruk AV, Butryojantho C, Jirasakonpat B, Junta N, Srichai S, Puapairoj C, Simtong P (2019). Phenotype frequencies of Rh (C, c, E, e), M, Mi a and Kidd blood
group systems among ethnic Thai blood donors from the north‐east of
Thailand. Int J Immunogenet.

[B34] Saleh RM, Zefarina Z, Mat NFC, Chambers GK, Edinur HA (2018). Transfusion medicine and molecular genetic
methods. Int J Prev Med.

[B35] Salzano FM (1964). Blood groups of Indians from Santa Catarina,
Brazil. Am J Phys Anthropol.

[B36] Salzano FM (1964). Demographic studies on Indians from Santa Gatarina,
Brazil. Acta Genet Med Gemellol.

[B37] Salzano FM, Callegari‐Jacques SM, Franco MHLP, Hutz MH, Weimer TA, Silva R, Da Rocha FJ (1980). The Caingang revisited: Blood genetics and
anthropometry. Am J Phys Antropol.

[B38] Salzano FM, Franco MHLP, Weimer TA, Callegari-Jacques SM, Mestriner MA, Hutz MH, Flowers NM, Santos RV, Coimbra CEA (1997). The Brazilian Xavante Indians revisited: New protein genetic
studies. Am J Phys Anthropol.

[B39] Suarez-Kurtz G, Pena SDJ, Struchiner CJ, Hutz MH (2012). Pharmacogenomic diversity among Brazilians: Influence of
ancestry, self-reported color, and geographical origin. Front Pharmacol.

[B40] Takezaki N, Nei M, Tamura K (2010). POPTREE2: Software for constructing population trees from allele
frequency data and computing other population statistics with Windows
interface. Mol Biol Evol.

[B41] Waskow G, Rodrigues MM de O, Höher G, Onsten T, Lindenau JD-R, Fiegenbaum M, Almeida S (2020). Genetic variability of blood groups in southern
Brazil. Genet Mol Biol.

[B42] Westhoff CM (2019). Blood group genotyping. Blood.

[B43] Zacarias JMV, Langer IBV, Visentainer JEL, Sell AM (2016). Profile of Rh, Kell, Duffy, Kidd, and Diego blood group systems
among blood donors in the Southwest region of the Paraná state, Southern
Brazil. Transfus Apher Sci.

