# Supplementary material for: Blood groups in Native Americans: a look beyond ABO and Rh
Source: Genet Mol Biol. 2021 Apr 19;44(2):e20200255. doi: 10.1590/1678-4685-GMB-2020-0255 (PMC8056887; doi:10.1590/1678-4685-GMB-2020-0255)
Supplement: Table S1 - [file 1415-4757-GMB-44-2-e20200255-s1.pdf]

Genetics and Molecular Biology

https://doi.org/10.1590/1678-4685-GMB-2020-0255

Supplementary Material to “Blood groups in Native Americans: a look beyond ABO and Rh”

Table 1 - Allele and estimated phenotype frequencies of antigens blood group in Kaingang and Guarani groups according to year of data collection.

Study/Blood group	Allele frequencies		Estimated phenotype frequencies[3]		
Diego	*DI*01*	*DI*02*	Di(a+b-)	Di(a+b+)	Di(a-b+)
Kaingang (1950-1960)[1]	0.236	0.764	0.056	0.361	0.583
Kaingang (2000)	0.118	0.882	0.014	0.208	0.778
Guarani (1950-1960)[2]	0.233	0.767	0.054	0.357	0.589
Guarani (1992-1993)	0.069	0.931	0.005	0.129	0.867
Kell	*KEL*01*	*KEL*02*	K+k-	K+k+	K-k+
Kaingang (1950-1960)[1]	0	1	0	0	1
Kaingang (2000)	0	1	0	0	1
Guarani (1950-1960)[2]	0	1	0	0	1
Guarani (1992-1993)	0.002	0.998	0	0.004	0.996
Duffy	*FY*01*	*FY*02*	Fy(a+b-)	Fy(a+b+)	Fy(a-b+)
Kaingang (1950-1960)[1]	0.710	0.290	0.504	0.412	0.084
Kaingang (2000)	0.576	4.242	0.332	0.488	0.180
Guarani (1950-1960)[2]	0.450	0.550	0.202	0.496	0.302
Guarani (1992-1993)	0.757	0.243	0.573	0.368	0.059
Kidd	*JK*01*	*JK*02*	Jk(a+b-)	Jk(a+b+)	Jk(a-b+)
Kaingang (1950-1960)[1]	0.629	0.371	0.396	0.467	0.137
Kaingang (2000)	0.674	0.326	0.455	0.439	0.106
Guarani (1950-1960)[2]	nt	nt	-	-	-
Guarani (1992-1993)	0.593	0.407	0.351	0.483	0.166

[1] Salzano et al. 1980; [2] Salzano 1964a. [3] Estimated phenotypes from the allele frequencies using the equation $p^2+{}^22pq+q^2=1$
